# Longitudinal cerebrospinal fluid measurements show glial hypo- and hyperactivation in predementia Alzheimer’s disease

**DOI:** 10.1186/s12974-023-02973-w

**Published:** 2023-12-13

**Authors:** Kaja Nordengen, Bjørn-Eivind Kirsebom, Grit Richter, Lene Pålhaugen, Berglind Gísladóttir, Nikias Siafarikas, Arne Nakling, Arvid Rongve, Geir Bråthen, Gøril Rolfseng Grøntvedt, Fernando Gonzalez, Knut Waterloo, Kulbhushan Sharma, Thomas Karikari, Eleonora M. Vromen, Betty M. Tijms, Pieter J. Visser, Per Selnes, Milicia G. Kramberger, Bengt Winblad, Kaj Blennow, Tormod Fladby

**Affiliations:** 1 Department of Neurology, Akershus University Hospital, P.B. 1000, 1478 Lørenskog, Norway; 2 Institute of Clinical Medicine, University of Oslo, Oslo, Norway; 3 Department of Neurology, University Hospital of North Norway, Tromsø, Norway; 4 Department of Psychology, Faculty Health Sciences, UiT, The Arctic University of Norway, Tromsø, Norway; 5 Clinical Molecular Biology (EpiGen), Medical Division, Akershus University Hospital and University of Oslo, Oslo, Norway; 6 Department of Old Age Psychiatry, Akershus University Hospital, Lørenskog, Norway; 7 Institute of Clinical Medicine, University of Bergen, Bergen, Norway; 8 Department of Research and Innovation, Haugesund Hospital, Helse Fonna, Haugesund, Norway; 9 Department of Clinical Medicine, University of Bergen, Bergen, Norway; 10 Department of Neuromedicine and Movement Science, Faculty of Medicine and Health Sciences, Norwegian University of Science and Technology, Trondheim, Norway; 11 Department of Neurology and Clinical Neurophysiology, University Hospital of Trondheim, Trondheim, Norway; 12 Clinical Neurochemistry Laboratory, Sahlgrenska University Hospital, Mölndal, Sweden; 13 Department of Psychiatry and Neurochemistry, The Sahlgrenska Academy at the University of Gothenburg, Mölndal, Sweden; 14 Department of Psychiatry, School of Medicine, University of Pittsburgh, Pittsburg, PA USA; 15 Alzheimer Center Amsterdam, Neurology, Vrije Universiteit Amsterdam, Amsterdam UMC Location Vumc, Amsterdam, the Netherlands; 16 Amsterdam Neuroscience, Neurodegeneration, Amsterdam, The Netherlands; 17 Department of Psychiatry, Maastricht University, Maastricht, the Netherlands; 18 Department of Neurology, University Medical Centre Ljubljana, Ljubljana, Slovenia; 19 Department of Neurobiology, Care Sciences and Society, Division of Clinical Geriatrics, Karolinska Institutet, Stockholm, Sweden; 20 Medical Faculty, University of Ljubljana, Ljubljana, Slovenia; 21 Department of Neurobiology, Care Sciences and Society, Division of Neurogeriatrics, Karolinska Institutet, Stockholm, Sweden

**Keywords:** Inflammation, Biomarkers, Cerebrospinal fluid, Alzheimer’s disease

## Abstract

**Background:**

Brain innate immune activation is associated with Alzheimer’s disease (AD), but degrees of activation may vary between disease stages. Thus, brain innate immune activation must be assessed in longitudinal clinical studies that include biomarker negative healthy controls and cases with established AD pathology. Here, we employ longitudinally sampled cerebrospinal fluid (CSF) core AD, immune activation and glial biomarkers to investigate early (predementia stage) innate immune activation levels and biomarker profiles.

**Methods:**

We included non-demented cases from a longitudinal observational cohort study, with CSF samples available at baseline (*n* = 535) and follow-up (*n* = 213), between 1 and 6 years from baseline (mean 2.8 years). We measured Aβ42/40 ratio, p-tau181, and total-tau to determine Ab (A+), tau-tangle pathology (T+), and neurodegeneration (N+), respectively. We classified individuals into these groups: A−/T−/N−, A+/T−/N−, A+/T+ or N+, or A−/T+ or N+. Using linear and mixed linear regression, we compared levels of CSF sTREM2, YKL-40, clusterin, fractalkine, MCP-1, IL-6, IL-1, IL-18, and IFN-γ both cross-sectionally and longitudinally between groups. A post hoc analysis was also performed to assess biomarker differences between cognitively healthy and impaired individuals in the A+/T+ or N+ group.

**Results:**

Cross-sectionally, CSF sTREM2, YKL-40, clusterin and fractalkine were higher only in groups with tau pathology, independent of amyloidosis (*p* < 0.001, A+/T+ or N+ and A−/T+ or N+, compared to A−/T−/N−). No significant group differences were observed for the cytokines CSF MCP-1, IL-6, IL-10, IL18 or IFN-γ. Longitudinally, CSF YKL-40, fractalkine and IFN-γ were all significantly lower in stable A+/T−/N− cases (all *p* < 0.05). CSF sTREM2, YKL-40, clusterin, fractalkine (*p* < 0.001) and MCP-1 (*p* < 0.05) were all higher in T or N+, with or without amyloidosis at baseline, but remained stable over time. High CSF sTREM2 was associated with preserved cognitive function within the A+/T+ or N+ group, relative to the cognitively impaired with the same A/T/N biomarker profile (*p* < 0.01).

**Conclusions:**

Immune hypoactivation and reduced neuron–microglia communication are observed in isolated amyloidosis while activation and increased fractalkine accompanies tau pathology in predementia AD. Glial hypo- and hyperactivation through the predementia AD continuum suggests altered glial interaction with Ab and tau pathology, and may necessitate differential treatments, depending on the stage and patient-specific activation patterns.

**Supplementary Information:**

The online version contains supplementary material available at 10.1186/s12974-023-02973-w.

## Background

Inflammatory activation has been proposed as a target for precision medicine therapy in Alzheimer’s disease (AD) and a detailed characterization of each pre-dementia stage is warranted [1]. AD stages may be described according to the core pathologies; amyloid plaques (A) and neurofibrillary tangles (T). Accompanying pathologies include neurodegeneration (N), vascular factors, synapse loss and inflammation. Genetic studies point to critical functions for microglia and macrophage-related mechanisms and transcription patterns in late onset AD (LOAD), interlinked with other AD pathologies [2, 3]. Neuron–glia communication is essential to uphold synaptic homeostasis and plasticity, and perivascular microglia, macrophages and astrocytes are linked to brain small-vessel and glymphatic function and homeostasis in, e.g., amyloid beta (Aβ)-clearance [4–6]. Cytokines are altered in neurodegenerative disease and AD, putatively reflecting compensatory or pathogenic mechanisms [7]. However, increased micro- and astroglial activation and inflammation in advanced AD are well-documented [7–10], and may be linked to synapse loss and neurotoxicity. Although the role of glial activation and the innate immune system in AD pathogenesis is multifaceted, neuroinflammation is generally considered detrimental, leading to proposals for trials with anti-inflammatory treatments [1, 11]. AD progression is associated with neurodegeneration (N) and has been described as an A/T/(N)-continuum [12, 13]. The A/T/(N) system allows use of cerebrospinal fluid (CSF) biomarkers to study stagewise associations of brain-derived cytokines previously linked to neuroinflammation and neurodegeneration.

The R47H variant of microglial membrane-bound triggering receptor expressed on myeloid cells 2 (*TREM2*) is a partial loss-of-function mutation and has been associated with a fourfold increase in the risk for LOAD. Partial loss-of-function (R47H variant) of the microglial membrane-bound triggering receptor expressed on myeloid cells 2 (TREM2) increases the risk for LOAD by fourfold, whereas common genetic variants associated with higher soluble TREM2 (sTREM2) levels are associated with lower LOAD risk and delayed age of onset [14, 15]. sTREM2 (shed from the membrane-bound form) activates microglia and increases fibrillar Aβ phagocytosis, whereas microglial membrane-bound TREM2 expression is linked to developmental synapse elimination [5, 14–17]. Uptake of Aβ by microglia is facilitated by the interaction between clusterin and APOE and is dependent on TREM2 [18]. Clusterin may also promote Aβ clearance [18, 19], and additionally contribute to increased complement activation and neuroinflammation [20–22], and *CLU* is a risk gene for LOAD. Neuron–microglia crosstalk is also subserved by the fractalkine (CX3CL1)/ CX3CR1 axis, which serves to uphold synaptic and neuronal homeostasis, to dampen inflammation but also subserve post-lesion and developmental synapse elimination in some systems [4, 5, 23]. Fractalkine and tau compete for binding at the microglial CX3CR1 receptor, which subserves microglial internalization of tau [24, 25]. YKL-40 (Chitinase-3-like protein) is involved in astro-microglial communication, and CSF-concentrations increase with tau-pathology and inflammation in AD [26, 27].

Cytokines such as interleukin 10 (IL-10) are involved in contact-independent microglia–neuron communication [28]. IL-10 has an anti-inflammatory effect and has been linked to an increase of dendritic spines. Single nucleotide polymorphisms (SNPs) both for IL-10, and for pro-inflammatory agents such as IL-6 and interferon gamma (IFN-g) have been associated with AD, and the latter two cytokines are also involved in neuron–microglia communication and may be linked to synapse loss [29, 30]. Pro-inflammatory IL-1 class cytokines such as IL-18 are generated by inflammasomes and can be induced by both tau and fibrillar Ab, as can monocyte chemoattractant protein-1 (MCP-1/CCL2) [31–33]. All are regulated by the Janus kinase (JAK)/signal transductor and activator of transcription proteins (STAT) pathway [34, 35].

Positron emission tomography (PET) studies employing ligands for translocator protein have reported a biphasic pattern of microglial activation, but the interpretation of these results is controversial [36–38]. CSF-based studies of glial activation might give more detailed information on net glial activation across the brain parenchyma. Several proteins related to immune/glia activation have been studied before, but longitudinal studies investigating CSF changes over time are limited [39].

Here, we employ a large longitudinal cohort with repeat CSF samples, studying markers for neuron- and glia communication and inflammation cross-sectionally, and in cases with stable A/T/N classification over time. Both the A−/T−/N−, the A+/T−/N− and the A+/T+ or N+ stages are well-defined and lengthy stages, avoiding transitional cases will provide a clearer picture of activation patterns and relevant targets for intervention at the respective stages [12, 40]. As a comparison to AD pathology, also cases with tau pathology, without amyloidosis, are included (A−/T+ or N+), known as suspected non-AD pathology (SNAP) [41, 42]. Comparing marker levels in cases on the AD continuum with cases with stable negative biomarkers and normal cognition, we ask how innate immune activation is linked to Ab and tau pathology at the main stages of the pre-dementia AD continuum, and in non-AD cases with abnormal tau markers.

## Materials and methods

### Study population

This study was a part of the Norwegian multi-center study Dementia Disease Initiation (DDI). The DDI cohort consists of non-demented individuals between 40 and 80 years of age primarily recruited from memory clinics and advertisements in local news media. For a detailed description of inclusion and exclusion criteria please see Fladby et al. [35]. We included a total of *n* = 535 participants who were recruited as either controls (*n* = 108) or reported Subjective Cognitive Decline (SCD, *n* = 207) or diagnosed with Mild Cognitive Impairment (MCI, *n* = 220). SCD was classified according to the SCD-I framework, which requires normal performance on neuropsychological tests while experiencing a subjective decline in any cognitive domain [36]. MCI was classified according to the National Institute on Aging and Alzheimer’s Association (NIA–AA) criteria, which requires the presence of subjective cognitive impairment or decline in combination with lower performance than expected in one or more cognitive domains, yet preserved independence in functional ability and not fulfilling the criteria of dementia [37]. Control cases reported no subjective cognitive decline and were recruited from spouses of patients with dementia/cognitive disorder, and patients who underwent orthopedic surgery with spinal anesthesia (and thus lumbar puncture). We determined the presence of cognitive impairment when results were 1.5 SD below the normative mean within one or more cognitive domains, including delayed memory recall [Consortium to Establish a Registry for Alzheimer’s Disease (CERAD) word list test] [38, 39], executive function [Trail Making Test part B (TMT-B)] [40], language/verbal fluency [Controlled Oral Word Association Test (COWAT)] [40, 41] and visuoperceptual ability (Visual Object and Space Perception Battery (VOSP) silhouettes) [42]. This procedure showed that *n* = 13 of 108 participants (12.04%) recruited as controls had scores consistent with possible MCI. A separate variable was computed based on the cognitive screening battery to account for this, where all participants recruited as controls with scores consistent with possible MCI and those with MCI were grouped as “MCI”, whereas controls and SCD with normal cognition were grouped as “cognitively normal” (CN). This variable was subsequently used for relevant statistical analyses.

#### Genetics, CSF collection, storage and analysis

Apolipoprotein E (*APOE*) genotyping was performed on EDTA blood samples as previously described [35]. Lumbar punctures were performed between 9 and 12 AM, and CSF samples were collected in sterile polypropylene tubes and centrifuged. CSF samples included prior to October 2020 used commercial enzyme-linked immunosorbent assays (ELISAs) from Innotest, Fujirebio, Ghent, Belgium based on monoclonal antibodies to determine CSF concentrations of total tau (t-tau, hTau Ag kits) and phosphorylated tau (p-tau, using 181P kits). Due to a change in laboratory equipment, CSF samples included after October 2020 used Elecsys t-tau and p-tau kits.

The QuickPlex SQ 120 system from Meso Scale Discovery (MSD, MD, USA) was used to measure Aβ_1–42_, Aβ_1–40_, YKL-40, clusterin, MCP-1, IL-6, IL-10, IL-18, fractalkine, IFN-γ and sTREM2. Aβ_1–42_ and Aβ_1–40_ was measured in a multiplex setup using V-plex Aβ Peptide Panel 1 (6E10) kit (K15200E-1). The samples were pre-diluted 1:2. YKL-40 was measured as single plex in a U-plex format and clusterin as single plex in a R-plex format, CSF samples were diluted 200 times prior to analyses for those analytes. IFN-γ and IL-10 were measured as single plex in a S-plex format and the samples were undiluted. MCP-1, IL-6, IL-18 and fractalkine were measured as a multiplex in a U-plex format, samples measured in 2017–2018 as a part of 9-Plex setup (100 μl neat CSF and 25 μl buffer per well) and samples analysed in 2020–2022 as 4-Plex setup (25 μl neat CSF and 25 μl buffer per well). sTREM2 was analyzed using a sandwich ELISA method described by others [43]. Briefly, the plate wells were blocked over-night, then coated with the capture antibody (0.25 μg/ml, biotinylated polyclonal goat IgG anti‐human TREM2, BAF1828, R&D Systems, MN, USA) for 1 h (shaking), prepared calibrator standards (recombinant human TREM2 protein, Hölzel Diagnostika) and samples were successively incubated (pre-diluted 1:4 with protease inhibitor to 0.25%) for 2 h (shaking), and then added TREM2 detection antibody (1 μg/ml, monoclonal mouse IgG anti‐human TREM2, sc-373828, Santa Cruz Biotechnology, CA, USA). Finally, a secondary antibody was added (0.5 μg/ml, sulfo‐tag‐labelled goat polyclonal anti‐mouse IgG, R32AC-5, Mesoscale Diagnostics, MD, USA) and the plate shaken in the dark for 1 h. In all the MSD analyses, the samples were analyzed in duplicates and reanalyzed if relative deviations (RDs) exceeded 20% and quality control samples with an RD threshold of 15% were controlled for interplate and interday variation. Due to differences in the 9-Plex and 4-plex setups for MCP-1, IL-6, IL-18 and fractalkine, we evaluated adjustments of between-setup differences in our statistical models (see “Statistical analyses” for details).

### A/T/N classification and study design

**Fig. 1:**
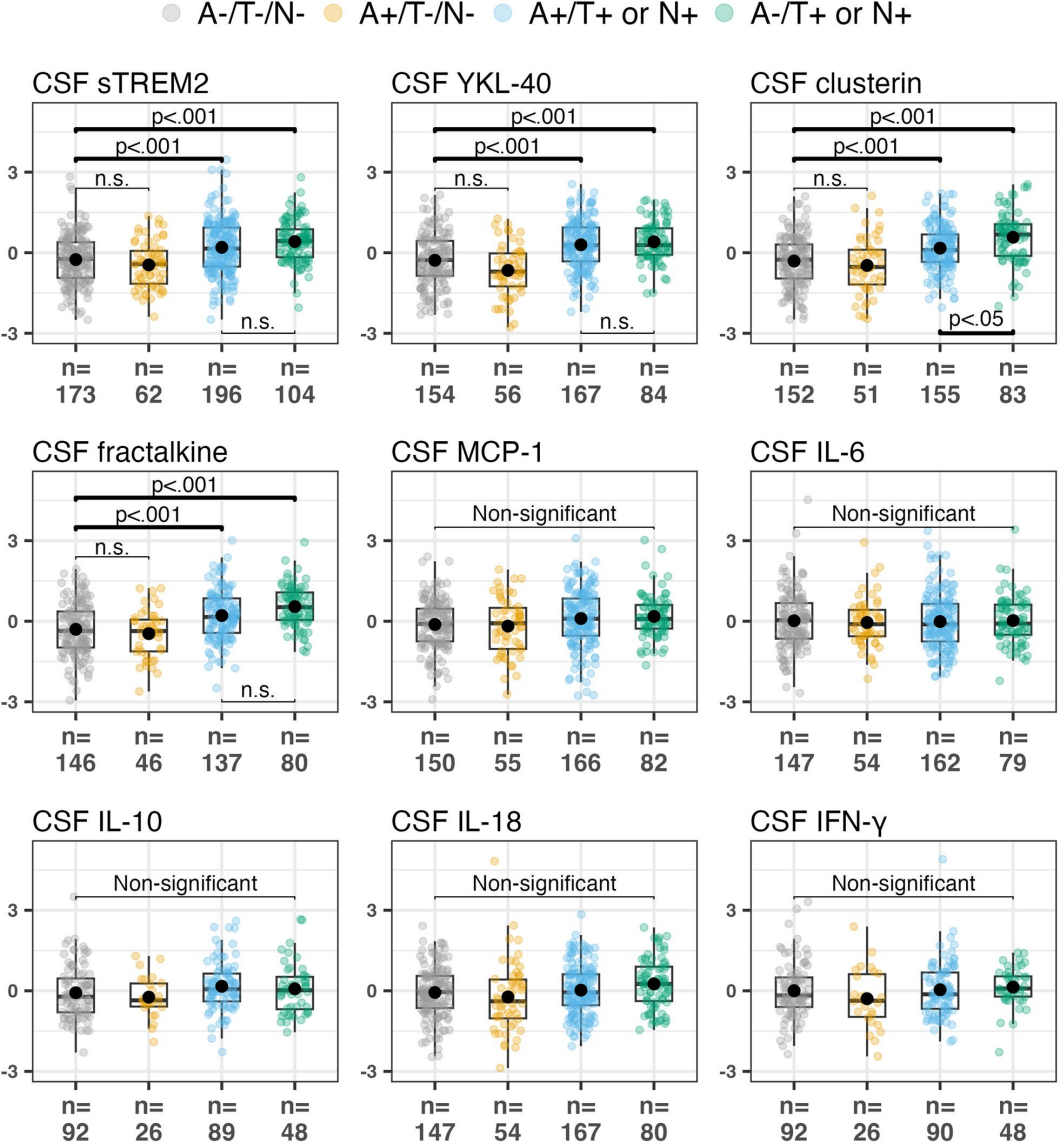
Cross-sectional comparisons between A/T/N groups for CSF immune biomarkers. Top row shows CSF sTREM2 YKL-40 and clusterin. The middle row shows fractalkine, MCP-1 and IL-6. The bottom row shows IL-10, IL-18 and IFN-γ. All *p* values are according to Bonferroni–Holm post-hoc adjustement

We used the A/T/N classification scheme [1] for biomarkers of hallmark Alzheimer’s disease pathology to determine the presence of amyloid plaques (A, CSF Aβ_42/40_ ratio), neurofibrillary tangles (T, CSF p-tau) and evidence of neurodegeneration (N, CSF t-tau) using CSF markers. The cutoff values for Innotest CSF t-tau and p-tau abnormality were applied according to unpublished cutoffs derived from receiver operating curve (ROC) analyses (Aβ-healthy controls vs. Aβ+ MCI/Dementia) within the DDI cohort (Innotest t-tau and p-tau) (≥ 378; ≥ 66.5); Elecsys t-tau and p-tau (≥ 228; ≥ 19). Please see Additional file 1: Table S1 for details on tau assay usage at baseline, and at follow-up. An optimum cutoff for mesocale Aβ_42/40_ ratio at ≤ 0.077 was determined following using ROC analysis using visual read of [18F]-Flutemetamol PET scans as the standard of truth. For a visualization of CSF Aβ_42/40_ ratio values within and between A/T/N-groups, please see our recent publication [44]. For the cross-sectional comparison of CSF immune activation markers, we selected four groups based on the following A/T/N staging: (a) cases with amyloid pathology without tau pathology (A+/T−/N−, *n* = 62), (b) cases with amyloid pathology and at least one pathological T or N marker (A+/T+ or N+, *n* = 196), (c) cases with evidence of tau-mediated neuropathology, but not amyloid pathology (A−/T+ or N+, *n* = 104) and (d) CN (recruited as controls or SCD) with normal CSF AD biomarkers (A−/T−/N−, *n* = 173). For the cross-sectional analyses, CSF immune marker availability varied between groups, but all sample sizes were adequate for statistical comparison (see Table 1 and Fig. 1 for details). For the longitudinal analyses, the same groups were selected based on stable CSF biomarker characteristics over time, where all cases included had at least one repeated CSF measure: (a) stable A+/T−/N− (*n* = 18), (b) stable A+/T+ or N+ (*n* = 89), (c) stable A−/T+ or N+ (*n* = 29) and (d) stable CN A−/T−/N− participants (*n* = 77). Here, most had immune markers available at all available timepoints within the groups. However, three sTREM2 measurements were missing in each of the pathological stable A/T/N groups and one stable A−/T+ or N+ case was missing a baseline measurement for YKL-40, fractalkine, MCP1, IL-6, IL-10, IL-18 and IFN-γ, but had repeated measures. A total of *n* = 213 had available CSF samples at least one follow-up assessment (*M* = 2.14 years, SD = 0.66), *n* = 74 had two follow-ups (*M* = 4.34 years, SD = 0.84) and *n* = 12 had three follow-ups (*M* = 5.62 years, SD = 0.59). The overall mean follow-up time was 2.83 years (SD = 1.31), ranging between 0.75 to 8.5 years from baseline (please see Additional file 1: Table S2 for detailed description of follow-up numbers and time within A/T/N groups). Please see Additional file 2: Figure S1 for details regarding T and/or N+ distributions within the T or N+  groups for both cross-sectional and longitudinal analyses.

**Table 1:** Between-group comparisons of baseline demographics, *APOE-ε4* carrier status and CSF immune markers

	ATN groups (*n*)					Statistical tests *p* (*p*.adj)			
	CN A−/T−/N− (173)	A+/T−/N− (62)	A+/T+ or N+ (196)	A−/T+ or N+ (104)	*F*/*χ*^2^/*η*^2^/*η*_*p*_^2^ *(p)*	A−/T−/N− vs. A+/T−/N−	A−/T−/N− vs. A+/T+ or N+	A−/T−/N− vs. A−/T+ or N+	A+/T+/N+ vs. A−/T+ or N+
Age mean (SD)	59.65 (8.80)	66.61 (8.12)	68.33 (7.44)	64.94 (9.88)	*F* = 32.34, *η*^*2*^ = 0.15 (< **0.001**)	^a^< 0.001 (< **0.001**)	^a^< 0.001 (< **0.001**)	^a^< 0.001 (< **0.001**)	^a^< 0.01 (< **0.05**)
Female n (%)	95 (54.91)	41 (66.13)	100 (51.02)	56 (53.85)	*χ*^*2*^ = 2.69, (0.452)	^c^	^c^	^c^	^c^
*APOE-ε4*+ *n* (%) [missing *n*]	59 (33.33) [*n* = 3]	44 (68.75) [*n* = 3]	142 (74.35) [*n* = 7]	39 (38.61) [*n* = 7]	*χ*^*2*^ = 77.22, (< **0.001**)	^c^	^c^	^c^	^c^
Recruited as controls *n* (%)	57 (32.95)	9 (14.52)	19 (9.69)	23 (22.12)		^c^	^c^	^c^	^c^
SCD *n* (%)	116 (67.05)	23 (37.09)	39 (19.90)	29 (27.88)		^c^	^c^	^c^	^c^
MCI *n* (%)	0 (0)	30 (48.39)	138 (70.41)	52 (50.00)		^c^	^c^	^c^	^c^
*CN *n* (%)/*MCI *n* (%)	173 (100)/0 (0)	29 (46.77)/33 (53.23)	51 (26.02)/145 (73.98)	49 (47.12)/55 (52.88)	**χ^*2*^ = 17.18, (< **0.001**)	^c^	^c^	^c^	^c^
sTREM2 mean (SD) [*n*]	3.71 (1.34) [173]	3.85 (1.17) [62]	5.06 (1.91) [196]	5.04 (1.69) [104]	*F* = 19.51, *η*_*p*_^2^ = 0.10, (< **0.001**)	^b^n.s. (n.s.)	^b^< 001 (**< 001**)	^b^ < 001 (**< 001**)	^b^n.s. (n.s.)
YKL-40 mean (SD) [*n*]	143.26 (57.30) [154]	145.07 (49.49) [56]	210.93 (69.10) [167]	197.34 (58.49) [84]	*F* = 28.07, *η*_*p*_^*2*^ = 0.16, (< **0.001**)	^b^n.s. (n.s.)	^b^ < 001 (**< 001**)	^b^ < 001 (**< 001**)	^b^n.s. (n.s.)
Custerin mean (SD) [*n*]	1865.34 (582.52) [152]	1889.75 (630.50) [51]	2420.87 (752.67) [155]	2665.40 (872.20) [83]	*F* = 25.10, *η*_*p*_^*2*^ = 0.15, (< **0.001**)	^b^n.s. (n.s.)	^b^ < 001 (**< 001**)	^b^ < 001 (**< 001**)	^b^ <0.05 (n.s.)
Fractalkine mean (SD) [*n*]	1848.57 (530.25) [146]	1810.54 (425.11) [46]	2256.21 (616.43) [137]	2379.51 (562.01) [80]	*F* = 21.23, *η*_*p*_^*2*^ = 0.14, (< **0.001**)	^b^n.s. (n.s.)	^b^ < 001 (**< 001**)	^b^ < 001 (**< 001**)	^b^n.s. (n.s.)
MCP-1 mean (SD) [*n*]	462.80 (109.86) [148]	476.01 (125.42) [54]	534.39 (152.82) [163]	516.18 (137.41) [80]	*F* = 2.63, *η*_*p*_^*2*^ = 0.02, (< **0.05**)	^b^n.s. (n.s.)	^b^ < 05 (n.s.)	^b^ < 05 (n.s.)	^b^n.s. (n.s.)
Il-6 mean (SD) [*n*]	1.66 (0.87) [147]	1.50 (0.62) [54]	1.57 (0.79) [162]	1.62 (0.74) [79]	*F* = 0.09, (0.996)	^**c**^	^**c**^	^**c**^	^**c**^
IL-10 mean (SD) [*n*]	78.83 (58.68) [92]	63.62 (27.73) [26]	80.87 (47.31) [89]	82.63 (55.58) [48]	*F* = 1.07, (0.363)	^c^	^c^	^c^	^c^
IL-18 mean (SD) [*n*]	5.76 (2.30) [147]	6.27 (4.72) [54]	6.61 (2.57) [167]	6.90 (2.69) [80]	*F* = 2.94 *η*_*p*_^*2*^ = .02, (< **0.05**)	^b^n.s. (n.s.)	^b^(n.s.) (n.s.)	^b^ < 05 (n.s.)	^b^n.s. (n.s.)
IFN-γ mean (SD) [*n*]	51.73 (57.30) [92]	41.85 (40.76) [26]	57.54 (125.31) [90]	46.19 (20.91) [48]	*F* = 1.07, (0.363)	^c^	^c^	^c^	^c^

A±, CSF positive or negative for amyloid plaques; T±, CSF positive or negative for tau-tangles; N±, CSF positive or negative marker for neurodegeneration; SD, standard deviation; n, number of cases; %, percentage; *F*, *F* statistic; *χ*^*2*^*,* chi square statistic; *η*^*2*^*,* eta-squared; *η*_*p*_^*2*^, partial eta-squared; vs., versus; SCD, Subjective Cognitive Decline; CN, Cognitively Normal; MCI, Mild Cognitive Impairment. Significant statistical tests (ANOVA or ANCOVA, or between group comparisons after correction for multiple testing) are indicated in bold

*Tally of CN and MCI according to our cognitive screening battery, regardless of recruitment as control or SCD status

**Chi-square analyses do not include the CN A-/T-/N/-group

^a^ANOVA post-hoc (Bonferroni–Holm)

^b^ANCOVA comparisons (Bonferroni–Holm)

^c^No post-hoc comparisons performed

### Statistical analyses

#### DDI cohort

All analyses were performed in Rstudio (R version 4.2.2) [45]. Cross-sectional between-group comparisons of continuous variables with assumed normal distributions were performed with ANOVA (age) or ANCOVA (CSF Immune biomarkers adjusted for covariates). Here, age, sex and *APOE-ε4* status were assessed, but only kept in the final models if the pertinent covariate explained a significant proportion of the variance (*α* < 0.05). Nominal variables sex, diagnostic group (CN and MCI), and *APOE-ε4* genotype were assessed with Chi-square tests. For ANOVA and ANCOVA, post-hoc comparisons with Bonferroni–Holm adjustments were applied for each model (6 tests per model). Effect sizes for ANOVA (eta-squared) and ANCOVA (partial eta-squared) are reported. Linear mixed models (LMMs) were used to assess longitudinal changes in CSF immune markers with the stable CN A−/T−/N− group as the reference, and all models were fitted with a random slope for time. Annual change over time for each group was also computed using the emmeans R package [46]. For each model, Bonferroni–Holm adjustments were applied (10 tests per model). For both cross-sectional and longitudinal analyses, age was standardized (z-standardization), and the CSF immune markers were log-transformed and standardized (z-log). Years from baseline was kept unstandardized for the longitudinal models. Owing to a difference in 4 plex vs. a 9 plex setup, (see CSF analysis section above for details) used for IL-6, IL-18, MCP-1 and fractalkine, a random intercept for setup differences was assessed in both the cross-sectional and longitudinal models. However, results and model fit did not change for the cross-sectional analyses, and these adjustments were only kept for the longitudinal models. Following results from our longitudinal models, we also assessed longitudinal differences in CSF sTREM2, YKL-40, clusterin, fractalkine and MCP-1 between CN and MCI cases within the stable A+/T+ or N+ group. This was not performed within the stable A+/T−/N− or stable A−/T+ or N+ groups, as observations split by CN/MCI status were deemed too low for statistical analysis (see Table 2). Finally, frequencies and percentages of clinical progression within the observational period (progression to MCI or progression to dementia) for each stable A/T/N group were computed. In addition, survival curves were plotted for overall clinical progression (MCI and dementia) over the observed follow-up period. Hazard ratios were not computed, since the reference group (A−/T−/N−) was selected as CN at baseline and over time (see Fig. 5). A complete account of clinical stability (cases remaining CN or MCI) or change (reverting from MCI to CN) is shown in Additional file 3: Figure S2. For ease of visual comparisons between models, plots were created with either covariate adjusted z-log values (cross-sectional models), or regression predicted z-log values (longitudinal models). Plots were created using the ggeffects, ggpubr and ggplot2 R packages [47–49].

**Table 2:** Between-group comparisons of baseline demographics, *APOE-ε4* carrier status in the longitudinal subsample

	ATN groups (*n*)					Statistical tests *p* (*p*.adj)			
	Stable CN A−/T−/N− (77)	Stable A+/T−/N− (18)	Stable A+/T+ or N+ (89)	Stable A−/T+ or N+ (29)	*F*/*χ*^*2*^/*η*^*2*^ (*p*)	A−/T−/N− vs. A+/T−/N−	A−/T−/N− vs. A+/T+ or N+	A−/T−/N− vs. A−/T+ or N+	A+/T+/N+ vs. A−/T+ or N+
Age mean (SD)	60.04 (8.83)	66.17 (8.34)	68.70 (6.78)	66.52 (9.10)	*F* = 16.56, *η*^*2*^ = 0.15 (< **0.001**)	^a^< 0.01 (< **0.05**)	^a^< .001 (< **0.001**)	^a^< .001 (< **0.001**)	^a^n.s (n.s.)
Female *n* (%)	42 (54.54)	14 (77.78)	43 (48.31)	14 (48.28)	*χ*^*2*^ = 5.55, (0.135)	^b^	^b^	^b^	^b^
APOE-ε4**+ ***n* (%) [missing *n*]	28 (36.36) [*n* = 0]	13 (72.22) [*n* = 0]	66 (74.16) [*n* = 1]	10 (34.48) [*n* = 0]	*χ*^*2*^ = 32.14, (< **0.001**)	^b^	^b^	^b^	^b^
Recruited as controls *n* (%)	25 (32.47)	1 (5.56)	10 (11.24)	4 (13.79)		^b^	^b^	^b^	^b^
SCD *n* (%)	52 (67.53)	7 (38.89)	23 (25.84)	13 (44.83)		^b^	^b^	^b^	^b^
MCI *n* (%)	0 (0)	10 (55.55)	56 (62.92)	12 (41.38)		^b^	^b^	^b^	^b^
*CN n (%)/*MCI *n* (%)	77 (100)/0 (0)	7 (38.89)/11 (61.11)	28 (31.46)/61 (68.54)	17 (58.62)/12 (41.38)	**χ^*2*^ = 8.90, (< **0.01**)	^b^	^b^	^b^	^b^

Significant statistical tests (ANOVA or ANCOVA, or between group comparisons after correction for multiple testing) are indicated in bold

A±, CSF positive or negative for amyloid plaques; T±, CSF positive or negative for tau-tangles; N±, CSF positive or negative marker for neurodegeneration; SD, standard deviation; n, number of cases; %, percentage; *F*, *F* statistic; *χ*^*2*^*,* chi square statistic; *η*^*2*^*,* eta-squared; vs., versus; SCD, Subjective Cognitive Decline; CN, Cognitively Normal; MCI, Mild Cognitive Impairment

*Tally of CN and MCI according to our cognitive screening battery, regardless of recruitment as control or SCD status

**Chi-square analyses do not include the CN A−/T−/N/− group

^a^ANOVA post-hoc (Bonferroni–Holm)

^b^No post-hoc comparisons performed

## Results

### A/T/N group differences in demographics

The CN A−/T−/N− participants were younger than the pathological A/T/N cases (between 5 and 8 years on average). The A+/T−N− (68.75%) and A+/T+ or N+ (74.35%) groups had higher frequencies of *APOE*-ε4 genotypes than the CN A−/T−/N− group (33.33%), whereas the A−/T+ or N+ (38.61%) had similar frequencies as the CN A−/T−/N− group. While we saw a generally higher percentage of females in the A+/T−/N− group (66.13%) than the other groups (between 51.02% and 54.91%), these differences were not statistically significant. Please see Table 1 for details. For the subsample of stable A/T/N groups used in the longitudinal analyses, the between-group differences were largely similar, but with an even larger percentage of females in the stable A+/T−N− subgroup (77.78%). Please see Table 2 for details.

### Relationships between CSF immune markers and the covariates APOE-ε4, age and sex in our models

There were weak, albeit significant associations between lower clusterin, fractalkine, IL-6 and IL-10 and *APOE*-ε4 genotype in the cross-sectional models, but only with IL-10 in the longitudinal models. Increasing age was significantly associated with higher CSF immune markers levels in both cross-sectional and longitudinal models, with the noted exceptions for IL-6, IL-10 and IFN-γ. Lower levels of MCP-1, IL-6, IL-18 and clusterin were found for females in the cross-sectional models, whereas in the longitudinal models, females had lower levels for MCP-1, IL-6 and IL-18, but not clusterin. Moreover, IFN-γ concentrations were higher for females in the longitudinal models. For a complete account of covariate associations to pertinent CSF immune markers, please see Additional file 1: Table S3A (cross-sectional models) and 3B (longitudinal models).

### Cross-sectional A/T/N group differences of CSF immune activation markers

We found that the cross-sectional concentrations of both sTREM2, YKL-40, fractalkine and clusterin were higher in the A+/T+ or N+ and A−/T+ or N+ groups as compared to A−/T−/N− (all *p* < 0.001). No significant differences in sTREM2, YKL-40, clusterin or fractalkine levels were found between A−/T−/N− and A+/T−/N−. While no differences in concentrations between A+/T+ or N+ and A−/T+ or N+ were demonstrated for sTREM2, YKL-40, clusterin concentrations were higher in A−/T+ or N+ cases as compared to A+/T+ or N+ (*p* < 0.05). Following post-hoc adjustments, no significant between-group differences were found for the other CSF immune markers (MCP-1, IL6, IL-10, Il-18 or IFN-γ). See Table 1 and Fig. 1 for details.

### Longitudinal trajectories of CSF immune markers in stable A/T/N groups

**Fig. 2:**
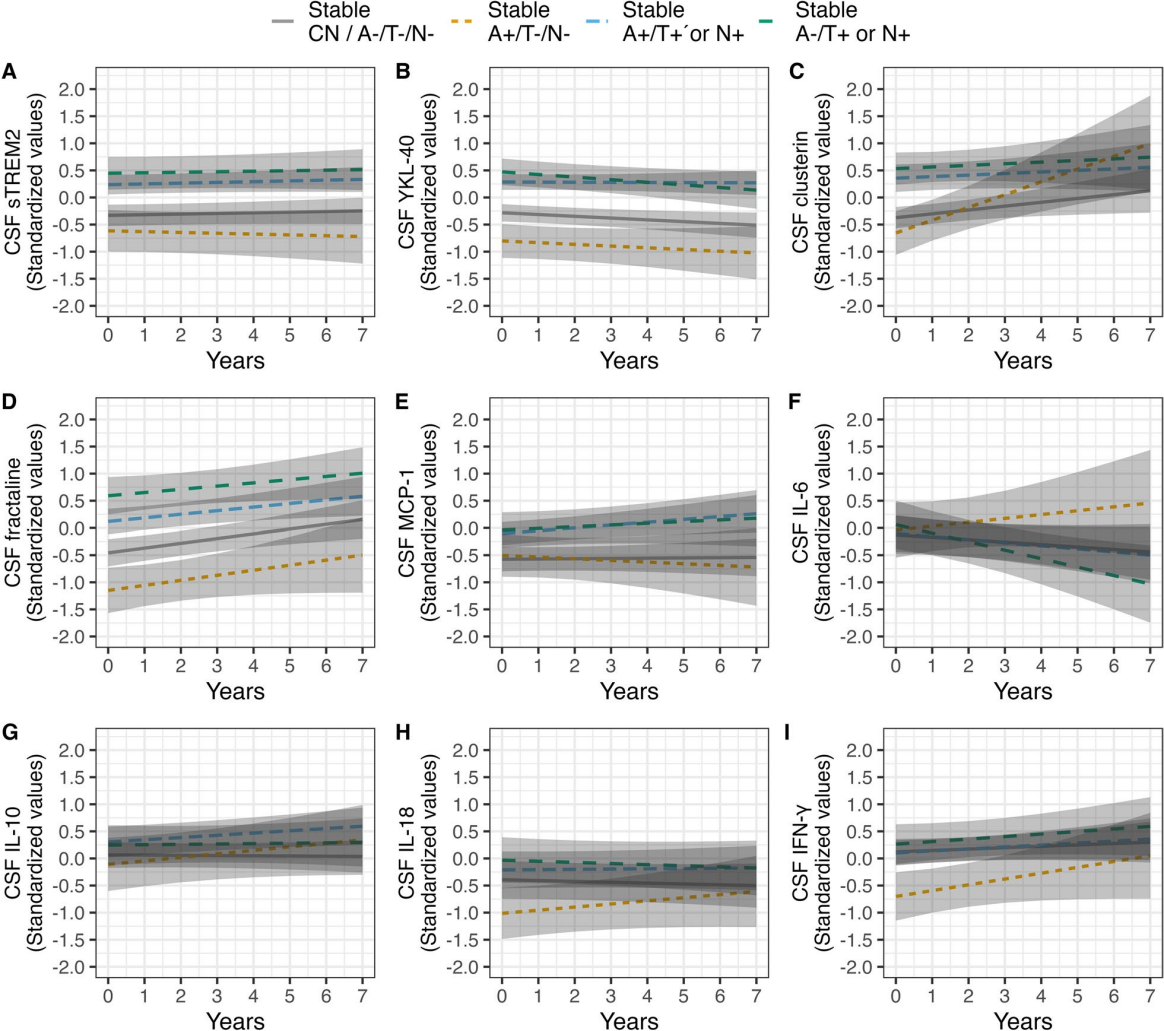
Between-group differences and longitudinal trajectories (up to 7 years) of CSF immune markers in stable CN A−/T−/N−, stable A+/T−/N−, stable A+/T+ or N+ and A−/T+ or N+ cases. The top row shows sTREM2 (**A**), YKL-40 (**B**), clusterin (**C**). The middle row shows fractalkine (**D**), MCP-1 and IL-6 (**F**). The bottom row shows IL-10 (**G**), IL-18 (**H**) and IFN-γ (**I**)

Biomarkers in clinically stable A-/T-/N-cases remained stable over time except for fractalkine that increased at follow-up (*p* < 0.05). Stable A+/T−/N− cases had lower baseline CSF YKL-40 (*p* < 0.05), fractalkine (*p* < 0.05) and IFN-γ (*p* < 0.05) concentrations than stable CN A−/T−/N−. The concentrations of these biomarkers remained stable over time. Moreover, while CSF IL-18 concentrations remained low over time in this group (unadjusted *p* < 0.01), this result was only at trend level following post-hoc adjustment for multiple testing (*p* = 0.064). While we observed an increase of clusterin concentrations over time (*p* < 0.01) in A+/T−/N− cases, this increase was not significantly different as compared to the stable CN A−/T−/N− cases. Both stable A+/T+ or N+ and A−/T+ or N+ had higher concentrations of sTREM2 (*p* < 0.001; *p* < 0.001), YKL-40 (*p* < 0.001; *p* < 0.001), clusterin (*p* < 0.001; *p* < 0.001), fractalkine (*p* < 0.001; *p* < 0.001) and MCP-1 (*p* < 0.05; *p* < 0.05) that all remained stable over time, see Table 3 and Fig. 2 for details.

**Table 3:** Longitudinal mixed linear models of CSF immune marker change by A/T/N group with stable A-/T-N-overtime as the reference group

CSF markers	Stable A−/T−/N− baseline (intercept)	Stable A−/T−/N− annual change	Stable A+/T−/N− baseline	Stable A+/T−/N− annual change	Stable A+/T+ or N+ baseline	Stable A+/T+ or N+ annual change	Stable A−/T+ or N+ baseline	Stable A−/T+ or N+ annual change
	*b* (95% CI)	*b* (95% CI) [*p*/*p*.adj]	*b* (95% CI) [*p*/*p*.adj]	*b* (95% CI) [*p*/*p*.adj]	*b* (95% CI) [*p*/*p*.adj]	*b* (95% CI) [*p*/*p*.adj]	*b* (95% CI) [*p*/*p*.adj]	*b* (95% CI) [*p*/*p*.adj]
sTREM2	− 0.33 (− 0.53; − 0.13)	− 0.01 (− 0.02; 0.39) [0.384/1.0]	− 0.29 (− 0.72; 0.15) [0.199/1.0]	− 0.01 (− 0.07; 0.04) [0.613/1.0]	0.57 (0.29; 0.85) [< 0.001/< **0.001**]	0.01 (− 0.01; 0.04) [0.311/1.0]	0.78 (0.41; 1.15) [< 0.001/ < **0.001**]	0.01 (− 0.03; 0.05) [0.662/1.0]
YKL-40	− 0.28 (− 0.44; − 0.12)	− 0.03 (− 0.06; − 0.01) [0.039/0.276]	− 0.52 (− 0.88; − 0.16 [< 0.01/< **0.05**]	− 0.03 (− 0.10; 0.37) [0.366/1.0]	0.57 (0.34; 0.80) [< 0.001/< **0.001**]	− 0.01 (− 0.03; 0.03) [0.886/1.0]	0.75 (0.46; 1.05) [< 0.001/< **0.001**]	0.05 (− 0.10; − 0.01) [< 0.05/0.276]
Clusterin	− 0.37 (− 0.57; − 0.18)	0.07 (0.01; 0.13) [0.028/0.198]	− 0.28 (− 0.71; 0.15) [0.196/0.978]	0.24 (0.10; 0.38) [< 0.001/< **0.01**]	0.73 (0.43; − 1.03) [< 0.001/< **0.001**]	0.03 (− 0.04; 0.09) [0.380/1.0]	0.91 (0.56; 1.25) [< 0.001/< **0.001**]	0.03 (− 0.07; 0.13) [0.529/1.0]
fractalkine	− 0.46 (− 0.77; − 0.14)	0.09 (0.03; 0.15) [< 0.01/< **0.05**]	− 0.69 (− 1.14; − 0.25) [< 0.01/< **0.05**]	0.09 (− 0.02; 0.21) [0.113/0.565]	0.58 (0.29; 0.86) [< 0.001/< **0.001**]	0.07 (0.01; 0.12) [< 0.05/.156]	1.05 (0.68; 1.42) [< 0.001/< **0.001**]	0.06 (− 0.02; 0.14) [0.156/0.626]
MCP-1	− 0.57 (− 0.80; − 0.35)	0.01 (− 0.06; − 0.07) [0.879/1.0]	0.07 (− 0.38; 0.52) [0.759/1.0]	− 0.03 (0.14; 0.08) [0.589/1.0]	0.47 (0.19; 0.76) [< 0.01/< **0.05**]	0.05 (− 0.01; 0.11) [0.100/0.800]	0.54 (0.16; 0.91) [< 0.01/< **0.05**]	0.03 (− 0.06; 0.12) [0.473/1.0]
IL-6	− 0.13 (− 0.67; 0.42)	− 0.05 (− 0.12; 0.04) [0.268/1.0]	0.09 (− 0.41; 0.59) [0.727/1.0]	0.07 (− 0.09; 0.23) [0.378/1.0]	0.03 (− 0.27; 0.32) [0.867/1.0]	− 0.06 (− 0.14; 0.02) [0.154/1.0]	0.19 (− 0.23; 0.60) [0.372/1.0]	− 0.16 (− 0.27; − 0.04) [< 0.05/0.095]
IL-10	0.06 (− 0.18; 0.31)	− 0.01 (− 0.05; − 0.04) [0.851/1.0]	− 0.18 (− 0.69; 0.34) [0.502/1.0]	0.07 (− 0.03; 0.16) [0.167/1.0]	0.24 (− 0.08; 0.56) [0.141/1.0]	0.04 (0.00; 0.08) [0.052/.518]	0.18 (− 0.24; 0.60) [0.395/1.0]	0.01 (− 0.06; 0.07) [0.829/1.0]
IL-18	− 0.39 (− 0.84; 0.06)	− 0.02 (− 0.06; 0.03) [0.500/1.0]	− 0.62 (− 1.06; − 0.18) [< 0.01/0.064]	0.06 (− 0.04; 0.15) [0.220/1.0]	0.18 (− 0.10; 0.46) [0.207/1.0]	0.01 (− 0.04; 0.05) [0.831/1.0]	0.36 (− 0.01; 0.73) [0.055/0.492]	− 0.02 (− 0.09; 0.05) [0.527/1.0]
IFN-γ	0.12 (− 0.12; 0.36)	0.03 (− 0.03; 0.09) [0.396/1.0]	− 0.82 (− 1.32; − 0.33) [< 0.01/ < **0.05**]	0.11 (− 0.02; 0.24) [0.107/0.967]	− 0.02 (− 0.31; 0.28) [0.898/1.0]	0.04 (− 0.02; 0.10) [0.238/1.0]	0.14 (− 0.27; 0.53) [0.499/1.0]	0.05 (− 0.04; 0.14) [0.298/1.0]

Significant statistical tests (ANOVA or ANCOVA, or between group comparisons after correction for multiple testing) are indicated in bold

A±, CSF positive or negative for amyloid plaques; T±, CSF positive or negative for tau-tangles; N±, CSF positive or negative marker for neurodegeneration; b, unstandardized regression coefficient; SE, standard error; *p*, *p* value; *p*.adj, Bonferroni–Holm adjusted *p* value. No significant between-group differences in slopes over time (Bonferroni–Holm) as compared with the reference group (stable A−/T−/N−) were found for any biomarker

### *Longitudinal trajectories of CSF sTREM2, YKL-40, clusterin, fractalkine and MCP-1 between CN and MCI cases in the stable A*+*/T or N*+ *group*

**Fig. 3:**
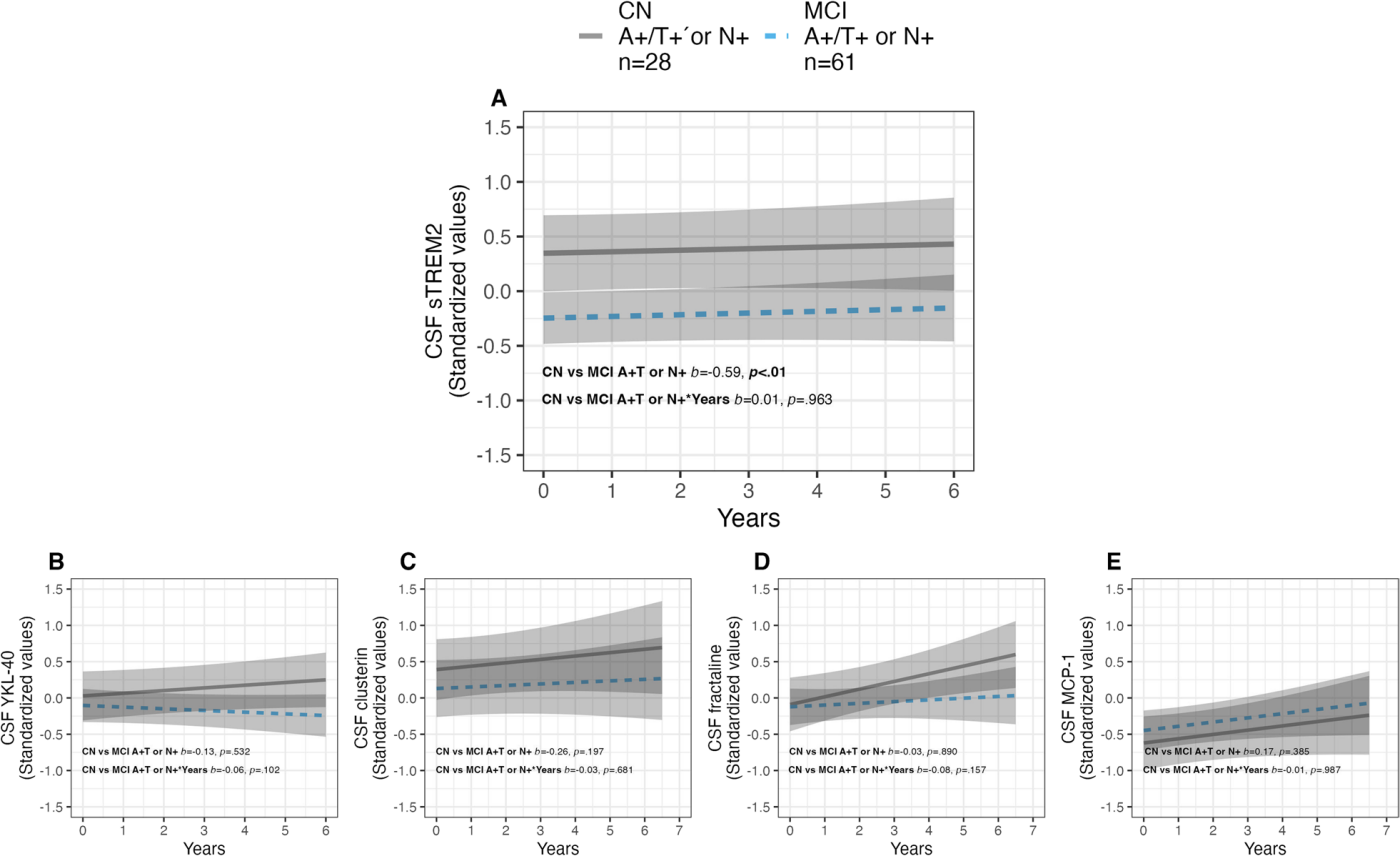
Longitudinal sub-analyses of CSF immune markers for CN vs. MCI within the A+/T+ or N+ group. The top figure (**A**) shows significantly higher sTREM2 values in the CN vs. the MCI group. The bottom row shows non-significant between-group differences for YKL-40 (**B**), clusterin (**C**), fractalkine (**D**) and MCP-1 (**E**)

Following the results from our main longitudinal analyses above, we decided to run sub-analyses between preclinical CN A+ cases and prodromal MCI cases within the stable A/T/N groups on markers that showed significant differences as compared to the CN A−/T−/N− group. However, due to the lower number of cases in stable A+/T−/N− and A−/T+ or N+, we were only able to run this analysis within the stable A+/T+ or N+ group. Here, we demonstrated higher sTREM2 levels in CN vs. MCI cases that remained stable during the follow-up period (*p* < 0.01). No significant between-group differences, or changes over time were found for the other markers (see Fig. 3 for details).

### Progression to MCI and dementia within the pathological A/T/N groups

**Fig. 4:**
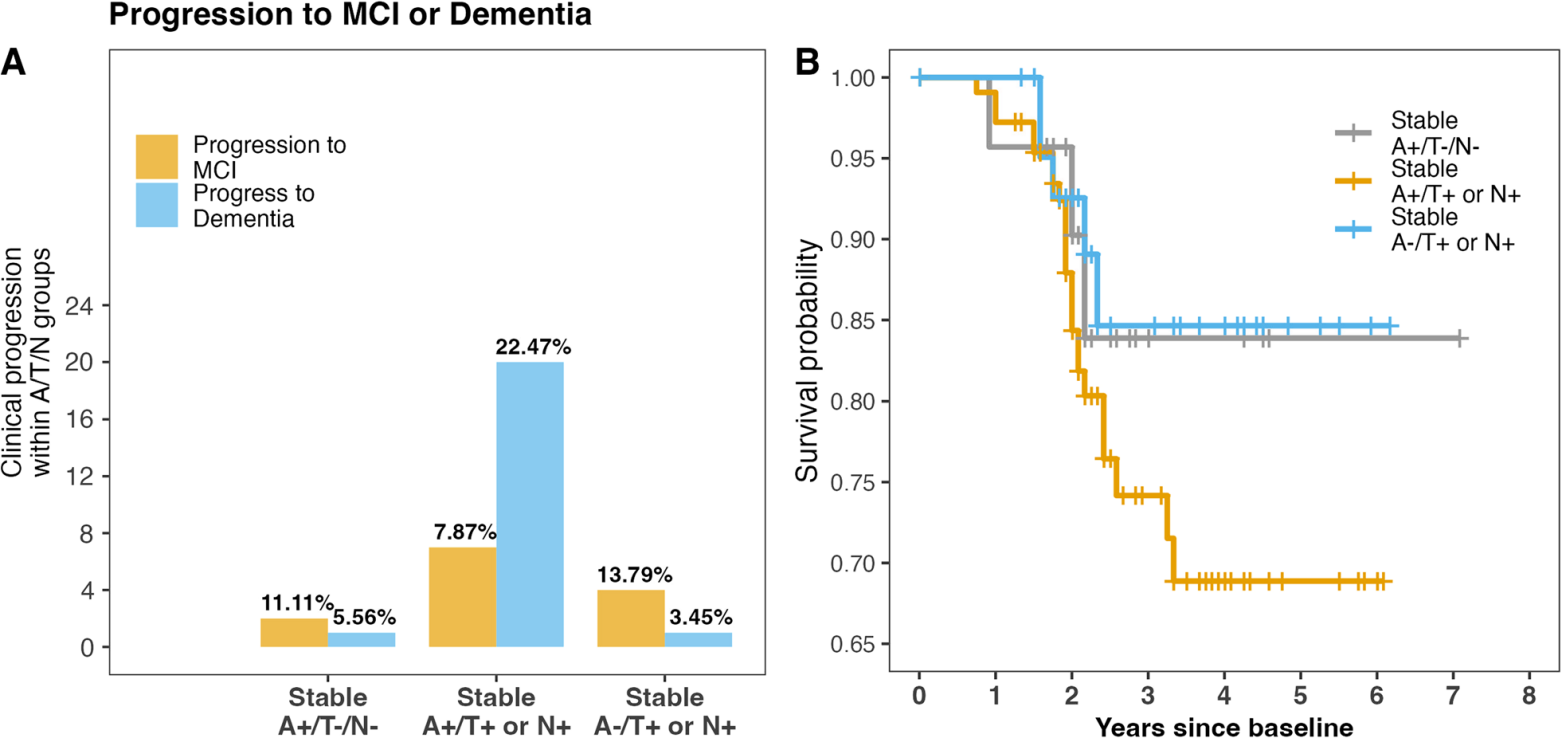
**A** Percentage of cases progressing to either MCI or dementia within pathological A/T/N groups during the observed follow-up period. **B** Survival curves of combined progression to MCI or dementia between the pathological A/T/N groups

During the follow-up period, *n* = 2 (11.11%) of the stable A+/T−/N− cases progressed to MCI, while *n* = 1 (5.55%) progressed to dementia. Within the stable A+/T+ or N+ group, *n* = 7 (7.86%) progressed to MCI, while *n* = 20 (22.47%) progressed to dementia. For the stable A−/T+ or N+ group, *n* = 4 (13.79%) progressed to MCI, while *n* = 1 (3.45%) progressed to dementia (see Fig. 4).

## Discussion

**Fig. 5:**
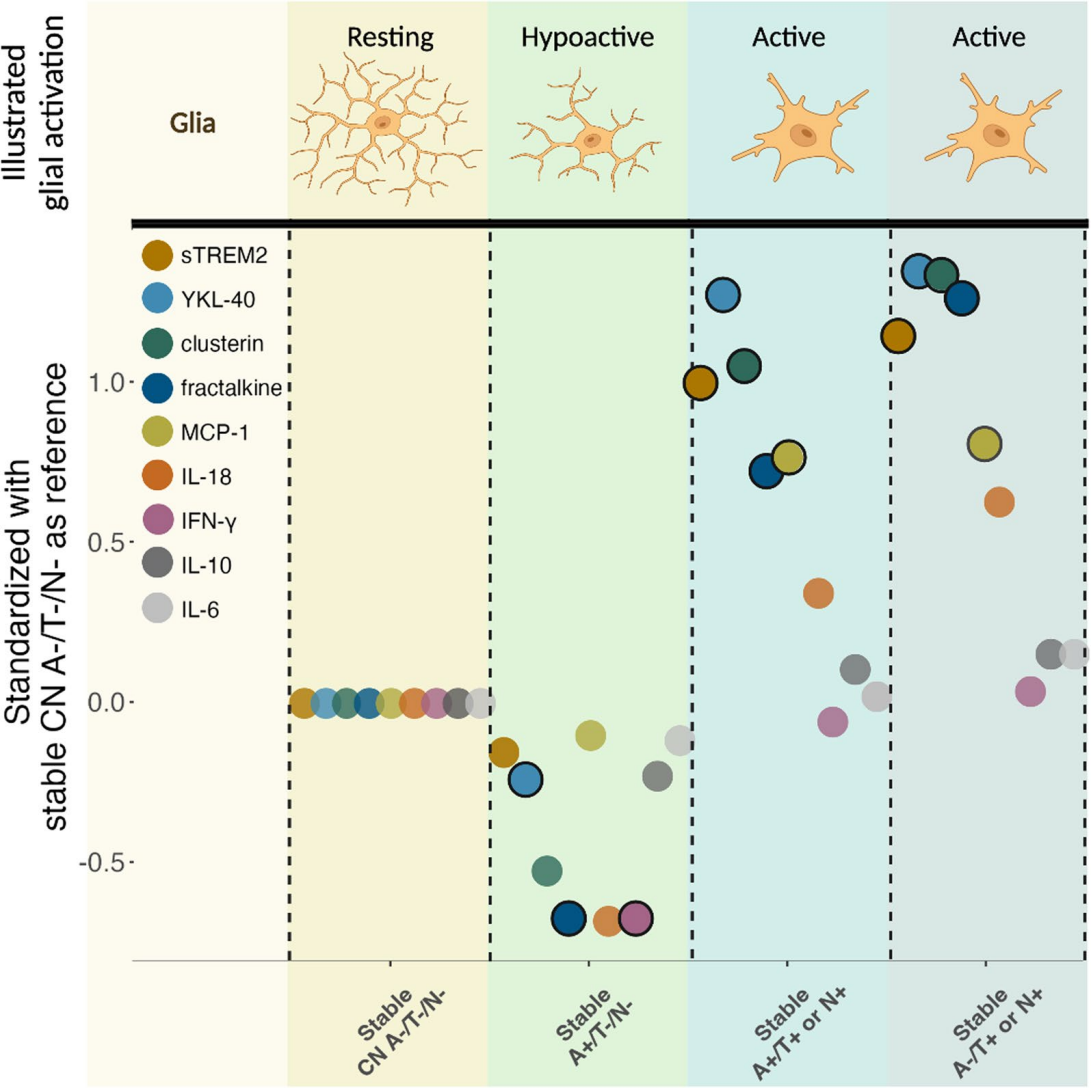
Graph illustrates CSF marker differences between stable A/T/N groups at baseline. All CSF markers are standardized with stable CN A−/T−/N− as the reference

In the present study, we found that biomarker stable A+/T−/N− cases had lower CSF baseline YKL-40, IFN-g and fractalkine levels compared to A−/T−/N− individuals. In those with tau pathology (T or N+), sTREM2, YKL-40, clusterin, and fractalkine were all significantly higher at baseline, regardless of amyloidosis (A− or A+). Within the A+/T+ or N+ group, we observed significantly higher levels of CSF sTREM2 in cognitively normal cases as compared to MCI. All the analyzed biomarkers remained stable over time, with the exception of clusterin which increased from baseline to follow-up in stable A+/T−/N−, and CSF fractalkine, which increased over time in all A/T/N groups (baseline results summarized in Fig. 5).

Glial activation is heterogenous with different states of activation related to brain physiology and local pathology [50]. Though dysfunctional support at the synapse has been proposed as a corollary to AD, we are not aware of earlier reports suggesting glial hypoactivation in incipient neurodegeneration (but see [51]). Our findings are further supported by gene ontology analysis, showing reduced gliogenesis in individuals with amyloidosis without tau pathology, and increased gliogenesis in individuals with both amyloid and tau pathology [52]. Microglial phagocytosis, autophagy and endolysosomal processing are prominent CNS innate immune functions linked to Aβ-metabolism at the synapse. LOAD innate-immune expressed risk genes are linked to these processes [11, 53, 54]. While our findings are based on a limited number of markers, lower levels of both YKL-40, IFN-g and fractalkine in stable A+/T−/N− support putative clinical relevance of an early stage of glial hypoactivation in A+/T−/N− cases. Of these, fractalkine and signaling via the fractalkine receptor CX3CR1 have been proposed as a promising target for treatment of neurodegenerative disease, including AD [5]. A pathomechanistic interpretation of these findings is underpinned by experimental findings in transgenic [PS1-APP-CX3CR1(±)] mice suggesting that suppressed fractalkine/CX3CR1 signaling may increase microglial phagocytosis and ameliorate Ab burden [55]. However, data from other transgenic mice lines (CX3CR1 (−/−, ±)) suggest that reduced neuron–microglial fractalkine signaling may be linked to reduced synaptic plasticity, reduced hippocampal neurogenesis and cognitive impairment [56]. The present findings illustrate how changes in key signaling molecules, such as fractalkine, may alleviate initial pathological changes, but also be linked to ensuing processes are detrimental for cognition and long-term neuronal viability.

Cell culture studies show that tau competes with fractalkine for uptake after binding to CX3CR1, and increased CX3CR1 expression in AD brains is associated with increased tau phosphorylation [24, 57]. Disturbed fractalkine/CX3CR1 signaling may alter tau phagocytosis, enhance formation of neurofibrillary tangles and contribute to a neurotoxic transformation of microglia [58, 59]. In line with this scenario, cases with stable tau pathology (A+/T+ or N+ and A−/T+ or N+) had sharply increased glial activation markers, including fractalkine. In these cases, increased phosphorylated tau is accompanied by increased fractalkine and increased expression of their common receptor CX3CR1. The concurrent marked increase in levels of sTREM2 and MCP-1, is expected to increase microglial phagocytic capacity, but at a cost of loss of homeostatic functions at the synapse. Phosphorylated tau shows reduced binding to CX3CR1, compatible with uncoupling of microglial activation/tau uptake and reduced microglial tau uptake at this stage [60]. Increased tau in brain interstitial fluid may propagate disease in connected areas and the formation of neurofibrillary tangles [61].

Most cases progressing to dementia were A+/T+ or N+ with fully developed AD pathology (Fig. 5). These findings are in line with in vivo observations that point to associations between microglial activation and tau pathology, and with recent large GWAS studies underscoring the interaction between microglial, Aβ and tau-linked pathways [62, 63]. In vivo PET studies also show stronger and more frequent association between microglial activation (measured with TSPO PET) and regional tau (measured with tau PET), than with regional Aβ (measured with amyloid PET) [64]. Here, they also found a positive correlation between CSF sTREM2 and tau PET in 4-repeat tau patients, but no correlation between CSF sTREM2 and amyloid PET [64]. Moreover, others have also reported elevated concentrations of CSF glial activation markers in SNAP cases (A−/T+) [65–67]. These findings are in accordance with the lack of glial activation in our cases with amyloid pathology alone (A+/T−/N−) and prominent glial activation in cases with tau pathology (A+/T+ or N+ or A−/T+ or N+). While recent experimental studies suggest that inflammation may proceed fibrillary tau pathology, these studies have used transgenic animal models [68, 69].

sTREM2 supports microglial Aβ phagocytosis, and increased CSF levels are associated with increased microglial activation in cross-sectional studies [16]. In transgenic animal models, amyloidosis results in more severe neurodegeneration and greater fibril branching in the absence of TREM2, suggesting a neuroprotective role [70, 71]. Though the role of TREM2 in humans is not fully understood, in a model using mice transgenic for a disease-associated human tau isoform with the P301s mutation, depletion of TREM2 (*Trem2*^−/−^) attenuates neuroinflammation and protects against neurodegeneration [72]. However, our finding that higher CSF sTREM2 levels were associated with preserved cognition in A+/T+ or N+ cases suggests a protective effect of TREM2 in humans. For CSF YKL-40, clusterin, fractalkine and MCP-1, high CSF levels are seen in cases with established tau-pathology (T+ or N+), regardless of amyloid status and seemingly independent of cognitive status, suggesting that links to tau, and not AD-specific pathology may be the main driver for these changes. Since microglia play a role in maintaining synaptic balance and promoting the elimination of unnecessary synapses, as well as in the regulation and clearance of Aβ and tau proteins, they may be involved in competing mechanisms that simultaneously aim to preserve synaptic structures and functions while also controlling Aβ and tau pathology.

For interleukins (IL-6, IL-10, IL-18) with pro- or anti-inflammatory properties, we neither found group-differences across A/T/N stages at baseline nor longitudinally, although a trend towards lower CSF IL-18 levels in isolated amyloidosis was observed which did not withstand correction for multiple comparisons. Though cases with tau pathology have significantly increased levels of glial activation markers, we do not see increased IL-18 levels, nor changes in the other inflammation linked cytokines (IL-10 and -6). Though this may be due to a limited set of markers, IL-18 is a key inflammasome activation marker, and we would expect to detect markedly increased inflammation [73]. Treatment of AD with anti-inflammatory drugs has so far not been successful [74, 75]. Our findings point to differential roles of innate immune activation and inflammation along the pre-dementia AD continuum, and effects of anti-inflammatory drugs may differ between disease subgroups and stages.

Finally, we included *APOE-ε4* genotype, age and sex as covariates in our statistical models. Of particular interest, females had lower levels of MCP-1, IL-6, IL-18 and clusterin than males in cross-sectional models, and all but clusterin in longitudinal models for cases which remained A/T/N stable over time. Moreover, IFN-γ concentrations were higher for females in the longitudinal models. However, because sex was only included as a covariate, we cannot tell whether these sex-differences might be differentially altered in the AD-continuum, or how differences could influence disease progression. Indeed, differential innate immune activation related to sex could influence disease–phenotype and progression in LOAD [63, 76, 77], and we are planning follow-up study to investigate the role of these sex differences on AD pathology and disease-progression.

This study has some limitations. First, dementia is the endpoint of the DDI longitudinal cohort study, and we do not have follow-up CSF data for cases after the onset of dementia. In contrast to most other AD cohorts, our cohort consists of relatively young, well-functioning individuals without dementia. Thus, evidence of further glia activation and inflammation at later stages of the AD clinical continuum cannot be ruled out. There is considerable heterogeneity in reported CSF alterations of immune activation ([9] Additional file 1: Table S1). This may be due to several factors, such as the clinical stage of disease, sample size and the inclusions of covariates in the models, such as age and sex. Our CSF cohort is comprehensive and carefully curated, which helps to reduce the variability often seen in results within this area of research. However, our sample size did not allow for analyses of clinical trajectories within or between A/T/N groups, except for A+/T+ or N+ group. However, we aim to pursue this question in more detailed analyses when more follow-up data are available. The CN A−/T−/N− group was significantly younger, but age was accounted for in all our statistical models. Finally, while our panel of markers is broad, it is not exhaustive and does not fully exclude early inflammatory engagement as it only covers a subgroup of possible mediators. However, our biomarker panel taps into the JAK/STAT-, inflammasome- and mitogen-activated protein kinases (MAPK) pathways [29, 78]. Innate immune signaling is mediated by several interlinked pathways, where nuclear factor kappa B (NF-kB) is a pivotal control complex and the mentioned pathways are major contributors [79]. Since activation of cytokines generally is network-driven and act in concert, we argue that a significant level of inflammation would be expected to be detected using this panel. Furthermore, these results are in accordance with recent neuropathological findings, also describing glial activation secondary to neurofibrillary pathology [80].

## Conclusions

Our findings add to the understanding of the early roles of microglia, and neuron–microglia communication in AD inception and development with novel longitudinal data pointing to early reduced activation and signaling coupled to Aβ and tau-linked pathological processes. These findings may have implications for AD therapy in that anti-inflammatory treatment may weaken glial activation at early susceptible stages. The roles of glial activation at different AD stages should be further explored, as should possibilities for selective targeting of inflammation vs. glial activation.

### Supplementary Information

**Additional file 1: Table S1.** Number and percentages of participants staged with p and t-tau using either Innotest or Elecsys assays. **Table S2.** Number of observations of CSF immune markers at each visit with follow-up time by visit split by A/T/N group. **Table S3A.** Covariate associations with cross-sectional CSF immune markers. **Table S3B.** Covariate associations with longitudinal CSF immune markers.

**Additional file 2: Figure S1**. Shows the T and/or N+ distributions within the T or N+ groups included in the cross-sectional (**A**) analyses, and longitudinal at baseline (**B**) and latest A/T/N measurement (**C**).

**Additional file 3: Figure S2.** Shows all clinical change or stability within pathological A/T/N groups during the follow-up time.

## Data Availability

Data from the DDI cohort are stored at Services for sensitive data (TSD) at the University of Oslo (UiO) and is publicly unavailable. However, anonymized data used in this study may be made available from the corresponding author upon reasonable request.
