# Supplementary material for: Longitudinal cerebrospinal fluid measurements show glial hypo- and hyperactivation in predementia Alzheimer’s disease
Source: J Neuroinflammation. 2023 Dec 13;20:298. doi: 10.1186/s12974-023-02973-w (PMC10720118; doi:10.1186/s12974-023-02973-w)
Supplement: Supplementary file 1 — Additional file 1: Table S1. Number and percentages of participants staged with p and t-tau using either Innotest or Elecsys assays. Table S2. Number of observations of CSF immune markers at each visit with follow-up time by visit split by A/T/N group. Table S3A. Covariate associations with cross-sectional CSF immune markers. Table S3B. Covariate associations with longitudinal CSF immune markers. [file 12974_2023_2973_MOESM1_ESM.docx]

**Additional tables**

| **Table S1.** Number and percentages of participants staged with p and t-tau using either Innotest or Elecsys assays | | | | | |
| --- | --- | --- | --- | --- | --- |
|  | Baseline (crossectional analysis) | Baseline (longitudinal analysis) | First follow-up (longitudinal analysis) | First follow-up (longitudinal analysis) | First follow-up (longitudinal analysis) |
| Innotest assay n(%) | 504 (94.2) | 213 (100) | 188 (88.3) | 60 (81.1) | 0 |
| Elecsys assay n(%) | 31 (5.8) | 0 (0) | 25 (11.7) | 14 (18.9) | 12 (100) |
| Abbreviations: n, number of cases; %, percentage. | | | | | |

| **Table S2.** Number of observations of CSF immune markers at each visit with follow-up time by visit split by A/T/N group | | | | | |
| --- | --- | --- | --- | --- | --- |
| **Groups** | **Baseline**  n | **First follow-up**  n (Mean Years) [SD] | **Second follow-up**  n (Mean Years) [SD] | **Third follow-up**  n (Mean Years) [SD] | **Total*** n (Mean Years) [SD] |
| **Stable  A-/T-/N-** | 77 | 77 (2.24)  [0.78] | 28 (4.13)  [0.32] | 6 (5.72)  [0.16] | 188 (2.91)  [1.25] |
| **Stable  A+/T-/N-** | 18 | 18 (2.15)  [0.49] | 5 (5.00)  [1.17] | 0 | 41 (2.77)  [1.37] |
| **Stable  A+/T+ or N+** | 89 | 89 (2.06)  [0.53] | 28 (4.31)  [0.81] | 4 (5.38)  [1.01] | 210 (2.69)  [1.24] |
| **Stable  A-/T+ or N+** | 29 | 29 (2.15)  [0.75] | 13 (4.62)  [1.33] | 2 (5.83)  [0.47] | 73 (3.05)  [1.59] |
| **Total** | 213 | 213 (2.14)  [0.66] | 74 (4.34)  [0.84] | 12 (5.62)  [0.59] | 512 (2.83)  [1.31] |
| Abbreviations: A+/-, Presence or absence of CSF Aβ_42/40_ ratio pathology; T+/-, Presence or absence of CSF p-tau pathology; T+/-, Presence or absence of CSF t-tau pathology; n, number of observations/cases; SD, standard deviation. Missing values: CSF sTREM2 (n=3); CSF YKL-40 (n=1); CSF IL-6 (n=7): CSF IL-10 (n=1); CSF IL-18 (n=1); CSF IFN-γ (n=1). *total numbers include baseline while mean follow-up time in years and SDs are computed for follow-ups. | | | | | |

| **Table S3A.** Covariate associations with cross-sectional CSF immune markers | | | |
| --- | --- | --- | --- |
| ***CSF marker***/ Predictor | **β** | **95% CI** | **p** |
| ***sTREM2*** | | | |
| APOE-e4 | -0.07 | -0.22, 0.09 | .40 |
| Age | 0.50 | 0.42, 0.58 | **<.001** |
| Male sex | 0.07 | -0.07, 0.21 | .30 |
| ***YKL-40*** | | | |
| APOE-e4 | -0.16 | -0.33, 0.02 | .075 |
| Age | 0.29 | 0.21, 0.38 | **<.001** |
| Male sex | -0.02 | -0.17, 0.13 | .80 |
| ***Clusterin*** | | | |
| APOE-e4 | -0.22 | -0.41, -0.04 | **.017** |
| Age | 0.26 | 0.17, 0.35 | **<.001** |
| Male sex | 0.24 | 0.08, 0.40 | **.003** |
| ***Fractalkine*** | | | |
| APOE-e4 | -0.23 | -0.43, -0.03 | **.024** |
| Age | 0.10 | 0.00, 0.20 | .052 |
| Male sex | -0.04 | -0.22, 0.14 | .60 |
| ***MCP-1*** | | | |
| APOE-e4 | -0.02 | -0.22, 0.18 | .90 |
| Age | 0.23 | 0.13, 0.33 | **<.001** |
| Male sex | 0.46 | 0.28, 0.63 | **<.001** |
| ***IL-6*** | | | |
| APOE-e4 | -0.22 | -0.43, -0.01 | **.045** |
| Age | 0.05 | -0.06, 0.15 | .40 |
| Male sex | 0.23 | 0.05, 0.42 | **.014** |
| ***IL-10*** | | | |
| APOE-e4 | -0.28 | -0.55, 0.00 | **.048** |
| Age | 0.07 | -0.07, 0.21 | .30 |
| Male sex | -0.06 | -0.31, 0.19 | .70 |
| ***IL-18*** | | | |
| APOE-e4 | -0.15 | -0.35, 0.05 | .14 |
| Age | 0.26 | 0.16, 0.36 | **<.001** |
| Male sex | 0.36 | 0.19, 0.54 | **<.001** |
| ***IFN-γ*** | | | |
| APOE-e4 | -0.10 | -0.38, 0.17 | .50 |
| Age | 0.12 | -0.02, 0.26 | .10 |
| Male sex | -0.17 | -0.42, 0.08 | .20 |
| Abbreviations: = β, standardized beta coefficient; CI, Confidence Interval; p, p-value. A/T/N groups were included in each model, but pertinent coefficients are only shown in table 1. | | | |

| **Table S3B.** Covariate associations with longitudinal CSF immune markers | | | |
| --- | --- | --- | --- |
| ***CSF marker***/ Predictor | **β** | **95% CI** | **p** |
| ***sTREM2*** | | | |
| APOE-e4 | -0.02 | -0.22, 0.19 | .90 |
| Age | 0.45 | 0.35, 0.55 | **<.001** |
| Male sex | 0.15 | -0.03, 0.33 | .10 |
| ***YKL-40*** | | | |
| APOE-e4 | -0.09 | -0.35, 0.17 | .50 |
| Age | 0.26 | 0.13, 0.39 | **<.001** |
| Male sex | 0.09 | -0.14, 0.31 | .50 |
| ***clusterin*** | | | |
| APOE-e4 | -0.21 | -0.44, 0.02 | .070 |
| Age | 0.18 | 0.07, 0.30 | **.002** |
| Male sex | 0.15 | -0.05, 0.35 | .14 |
| ***fractalkine*** | | | |
| APOE-e4 | -0.05 | -0.29, 0.19 | .70 |
| Age | 0.16 | 0.04, 0.27 | **.011** |
| Male sex | -0.07 | -0.27, 0.14 | 0.5 |
| ***MCP-1*** | | | |
| APOE-e4 | 0.01 | -0.24, 0.27 | .90 |
| Age | 0.16 | 0.03, 0.28 | **.017** |
| Male sex | 0.50 | 0.27, 0.72 | **<.001** |
| ***IL-6*** | | | |
| APOE-e4 | -0.17 | -0.43, 0.08 | .20 |
| Age | 0.06 | -0.07, 0.19 | .30 |
| Male sex | 0.29 | 0.06, 0.51 | **.012** |
| ***IL-10*** | | | |
| APOE-e4 | -0.35 | -0.63, -0.07 | **.015** |
| Age | 0.09 | -0.06, 0.23 | .20 |
| Male sex | -0.15 | -0.40, 0.10 | .20 |
| ***IL-18*** | | | |
| APOE-e4 | 0.05 | -0.20, 0.30 | .70 |
| Age | 0.20 | 0.07, 0.32 | **.002** |
| Male sex | 0.59 | 0.37, 0.81 | **<.001** |
| ***IFN-γ*** | | | |
| APOE-e4 | -0.11 | -0.38, 0.15 | .40 |
| Age | 0.08 | -0.06, 0.21 | .30 |
| Male sex | -0.28 | -0.51, -0.04 | **.020** |
| Abbreviations: = β, standardized beta coefficient; CI, Confidence Interval; p, p-value. A/T/N groups were included in each model, but pertinent coefficients are only shown in table 2. | | | |
