# Supplementary figures and images for: Longitudinal cerebrospinal fluid measurements show glial hypo- and hyperactivation in predementia Alzheimer’s disease
Source: J Neuroinflammation. 2023 Dec 13;20:298. doi: 10.1186/s12974-023-02973-w (PMC10720118; doi:10.1186/s12974-023-02973-w)

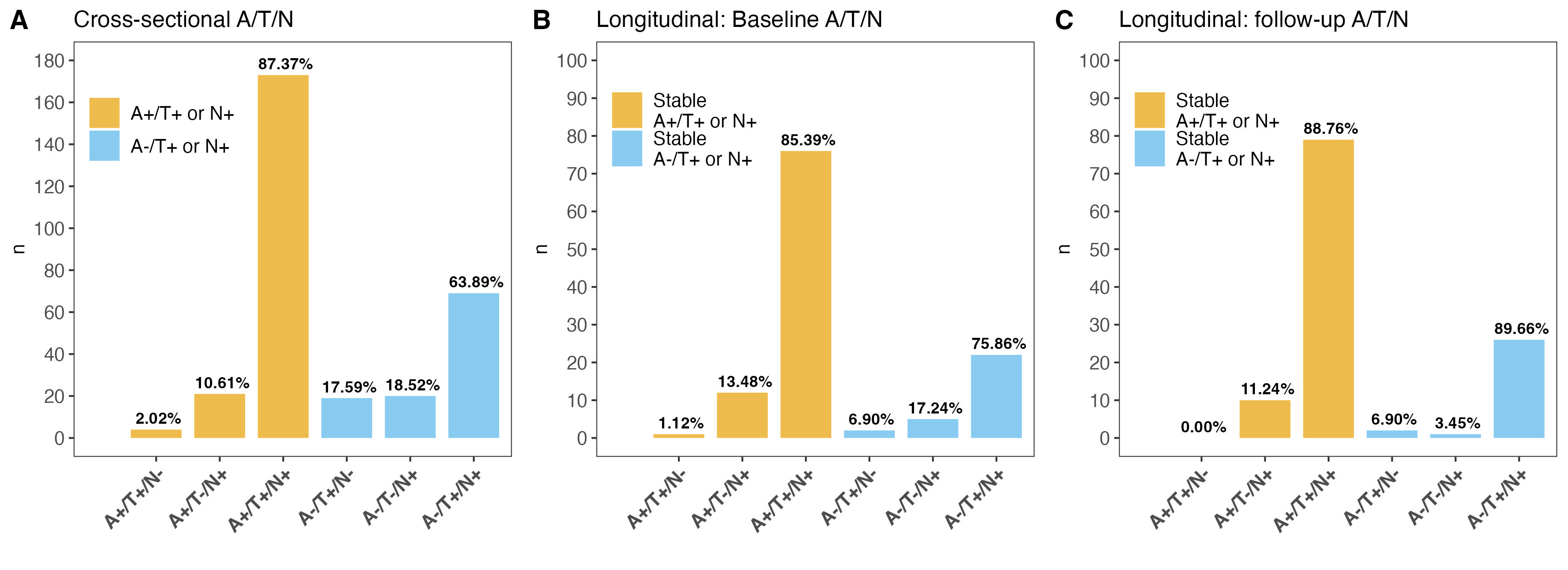

Supplement: Supplementary file 2 — Additional file 2: Figure S1. Shows the T and/or N+ distributions within the T or N+ groups included in the cross-sectional (A) analyses, and longitudinal at baseline (B) and latest A/T/N measurement (C). [file 12974_2023_2973_MOESM2_ESM.jpg]

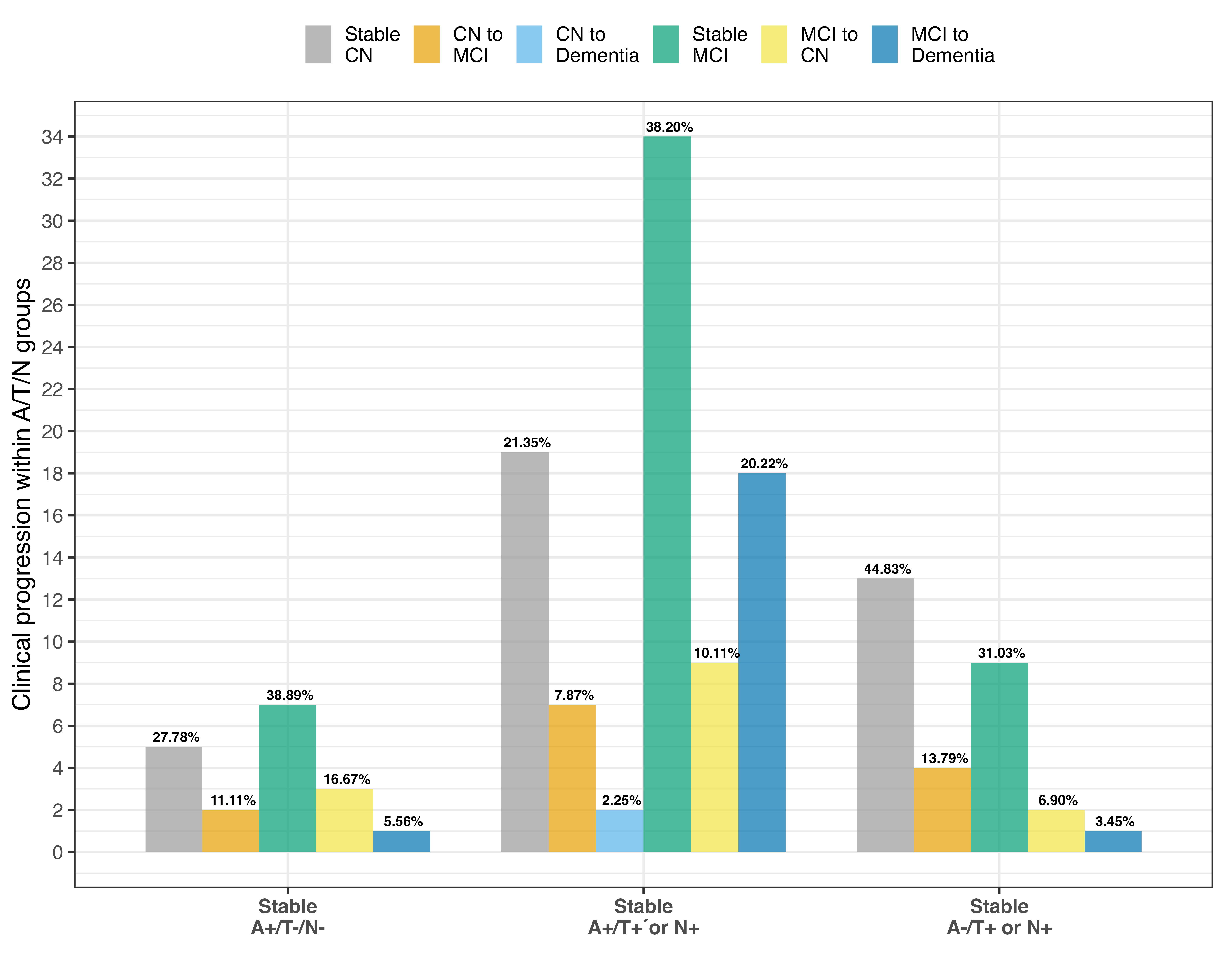

Supplement: Supplementary file 3 — Additional file 3: Figure S2. Shows all clinical change or stability within pathological A/T/N groups during the follow-up time. [file 12974_2023_2973_MOESM3_ESM.jpg]
